# In Vitro Antibody-Dependent Enhancement of SARS-CoV-2 Infection Could Be Abolished by Adding Human IgG

**DOI:** 10.3390/pathogens12091108

**Published:** 2023-08-30

**Authors:** Xun Wang, Minghui Li, Panpan Lu, Chen Li, Chaoyue Zhao, Xiaoyu Zhao, Rui Qiao, Yuchen Cui, Yanjia Chen, Jiayan Li, Guonan Cai, Pengfei Wang

**Affiliations:** 1 Shanghai Pudong Hospital, Fudan University Pudong Medical Center, Shanghai Institute of Infectious Disease and Biosecurity, State Key Laboratory of Genetic Engineering, MOE Engineering Research Center of Gene Technology, School of Life Sciences, Fudan University, Shanghai 200438, China; wang_xunfudan@fudan.edu.cn (X.W.); 21210700112@m.fudan.edu.cn (M.L.); lc876278963@163.com (C.L.); chaoyuejaul@163.com (C.Z.); xiaoyu_zhao@fudan.edu.cn (X.Z.); 21110700072@m.fudan.edu.cn (R.Q.); 21210700060@m.fudan.edu.cn (Y.C.); 22210700006@m.fudan.edu.cn (Y.C.); 22210700117@m.fudan.edu.cn (J.L.); caiguonan480@gmail.com (G.C.); 2 Reproductive Center, Women and Children’s Hospital, Qingdao University, Qingdao 266001, China; pplu16@fudan.edu.cn

**Keywords:** antibody-dependent enhancement, COVID-19, SARS-CoV-2, vaccination serum, convalescent serum

## Abstract

Evidence of antibody-dependent enhancement (ADE) of other viruses has raised concerns about the safety of SARS-CoV-2 vaccines and antibody therapeutics. In vitro studies have shown ADE of SARS-CoV-2 infection. In this study, we also found that vaccination/convalescent sera and some approved monoclonal antibodies can enhance SARS-CoV-2 infection of FcR-expressing B cells in vitro. However, the enhancement of SARS-CoV-2 infection can be prevented by blocking Fc–FcR interaction through the addition of human serum/IgG or the introduction of mutations in the Fc portion of the antibody. It should be noted that ADE activity observed on FcR-expressing cells in vitro may not necessarily reflect the situation in vivo; therefore, animal and clinical data should be included for ADE evaluation.

## 1. Introduction

The COVID-19 pandemic, caused by severe acute respiratory syndrome coronavirus-2 (SARS-CoV-2), has spread to over 200 countries, causing widespread morbidity and mortality, as well as massive economic losses [1,2]. To control the pandemic and prevent the recurrence of SARS-CoV-2, several vaccines designed by different platforms have been developed and approved, in addition to other immediate treatments, such as antibody-based therapies [3,4]. However, one of the biggest safety concerns with vaccines and antibody-based therapeutics is the antibody-dependent enhancement (ADE) of viral infections [5,6,7,8,9].

SARS-CoV-2 relies on angiotensin-converting enzyme 2 (ACE2) as its primary cell surface receptor to enter host cells. Neutralizing antibodies can effectively block the entry of the virus by inhibiting the binding of the SARS-CoV-2 Spike (S) to ACE2 [10,11]. However, certain antibodies could bind to Fc receptors (FcRs) on immune cells and be internalized, leading to an enhancement in virus entry [12]. This ADE phenomenon has been observed in the past with SARS-CoV, MERS-CoV, dengue virus (DENV), respiratory syncytial virus (RSV), and measles [13,14,15,16,17,18], raising concerns about the risk of ADE for SARS-CoV-2 vaccines and antibody-based therapies. Due to the great similarity between several bat coronaviruses and SARS-CoV-2, previous exposure to such viruses may lead to ADE of SARS-CoV-2 [19,20]. Higher antibody titers in patients with SARS-CoV-2 infection have been reported to associate with more severe disease, suggesting a possible link with ADE [21]. Several in vitro studies have demonstrated that the ADE infection of SARS-CoV-2 [22,23,24] and the emergence of new SARS-CoV-2 variants may increase the likelihood of ADE [25].

However, it remains unclear if in vitro ADE of infection can accurately predict enhanced infection in vivo. Using FcR-expressing B cells, we also observed enhanced viral infections induced by vaccination/convalescent sera and monoclonal antibodies (mAbs) in vitro. However, it is important to note that this ADE phenomenon could be eliminated through the addition of human serum/IgG or the introduction of mutations in the antibody’s Fc region. These results suggest that the observed in vitro ADE may not be a true predictor of ADE in real-life scenarios in the complex in vivo environment.

## 2. Materials and Methods

### 2.1. Cell Lines

Expi293F cells (Thermo Fisher, Waltham, MA, USA, Cat# A14527) were cultured in the serum-free SMM 293-TI medium (Sino Biological Inc., Beijing, China) at 37 °C with 8% CO_2_ on an orbital shaker platform. HEK293T cells (Cat# CRL-3216) and Vero-E6 cells (cat# CRL-1586) were acquired from ATCC and cultured in 10% Fetal Bovine Serum (FBS, GIBCO cat# 16140071) supplemented with Dulbecco’s Modified Eagle Medium (DMEM, ATCC cat# 30-2002) at 37 °C, 5% CO_2_. Raji cells (Cat# CCL-86) and THP-1 cells (cat# TIB-202) were acquired from ATCC and cultured in 10% FBS supplemented with Roswell Park Memorial Institute (RPMI) 1640 medium (Thermo Fisher, cat# 31870-082) at 37 °C, 5% CO_2_. I1 mouse hybridoma cells (ATCC, cat# CRL-2700) were cultured in Eagle’s Minimum Essential Medium (EMEM, ATCC cat# 30-2003) with 20% FBS.

### 2.2. Serum Samples

Sera from 7 individuals who received three doses of inactivated vaccine, or 7 individuals who were infected with the BA.5 variant after receiving three doses of inactivated vaccine, were recruited at the Nanjing Hospital of Chinese Medicine. For all COVID-19 participants, the clinical diagnosis criteria were based on the ninth National COVID-19 guidelines. The SARS-CoV-2 infection of all the subjects was confirmed by polymerase chain reaction (PCR) and sequencing. All participants involved in this study showed mild symptoms, or were asymptomatic. Two healthy individuals with no history of vaccination or infection were enrolled before the onset of the COVID-19 pandemic as controls at Huashan Hospital, Fudan University.

### 2.3. Monoclonal Antibodies

Monoclonal antibodies tested in this study were constructed and produced at Fudan University. For each antibody, variable genes were optimized for human cell expression and synthesized by HuaGene^TM^ (Shanghai, China). VH and VL were inserted separately into plasmids (gWiz or pcDNA3.4) that encode the constant region for the H chain and L chain. Monoclonal antibodies were expressed in Expi293F (Thermo Fisher, A14527) by co-transfection of the H chain and L chain expressing plasmids using polyethylenimine and cultured at 37 °C with shaking at 125 rpm and 8% CO_2_. On day 5, antibodies were purified using MabSelectTM PrismA (Cytiva, Marlborough, MA, USA, 17549801) affinity chromatography.

### 2.4. Construction and Production of Variant Pseudoviruses

Plasmids encoded with the WT (D614G) SARS-CoV-2 spike and Omicron XBB.1.5 spike were constructed. HEK293T cells were transfected with the indicated spike gene using polyethylenimine (Polyscience). Cells were cultured overnight at 37 °C with 5% CO_2_, and VSV-G pseudo-typed ΔG-luciferase (G*ΔG-luciferase, Kerafast, Winston-Salem, NC, USA) was used to infect the cells in DMEM at a multiplicity of infection of 5 for 4 h before washing the cells with 1 × DPBS three times. The next day, the transfected supernatant was collected and clarified by centrifugation at 3000× *g* for 10 min. Each viral stock was then incubated with 20% I1 hybridoma (anti-VSV-G; ATCC, CRL-2700) supernatant for 1 h at 37 °C to neutralize the contaminating VSV-G pseudotyped ΔG-luciferase virus before measuring titers and making aliquots to be stored at −80 °C.

### 2.5. Pseudovirus Neutralization Assays

Neutralization assays were performed by incubating pseudoviruses with serial dilutions of monoclonal antibodies or serum, and scored by the reduction in luciferase gene expression. In brief, Vero-E6 cells were seeded in a 96-well plate at a concentration of 2 × 10^4^ cells per well. A total of 750 TCID_50_ pseudoviruses were incubated the next day with serial dilutions of the test samples in triplicate for 60 min at 37 °C. The mixture was added to cultured cells and incubated for an additional 24 h. The luminescence was measured by the Luciferase Assay System (Beyotime, Shanghai, China). IC_50_ was defined as the dilution that resulted in a 50% reduction in relative light units compared to the control wells containing only the virus and cells. This measurement was taken after subtracting the background observed in control wells with cells alone. The IC_50_ values were calculated using nonlinear regression in GraphPad Prism.

### 2.6. Antibody-Dependent Enhancement (ADE) Assay

The ADE assays were performed using Raji cells. A total of 50 µL of serially diluted mAbs or mAbs combinations were mixed with 50 µL of supernatant containing 750 TCID_50_ of pseudovirus. The mixture was incubated for 60 min at 37 °C, and then supplied with 5% CO_2_. A total of 100 µL cells at a density of 2 × 10^6^ cells/mL were added to the mixtures of pseudoviruses and mAbs for an additional 24 h incubation. Then, the same volume of luciferase-detecting regents (Beyotime) was added to each well. After 2 min of incubation, the luciferase activity was measured by the Luciferase Assay System (Beyotime). The maximum ADE infectivity fold-change was calculated by comparing the peak ADE luciferase levels to those in the virus control wells (containing the virus and cells). This calculation was made after subtracting the background levels observed in the control groups containing cells only.

### 2.7. Generation of Mutated mAbs

For preparation of mutated mAbs to abolish their binding to FcRs, the heavy chain L234A/L235A combined mutations (LALA) were introduced to the parental hIgG1 mAbs. For preparation of mutated mAbs with increased binding to FcγRIIB, the heavy chain G117A, S120D, A211L, and I213E combined mutations (GASDALIE) were introduced to the parental hIgG1 mAbs. Mutated mAbs were expressed and purified by the method described above. Primer sequences used for cloning and mutations are listed in Table 1.

### 2.8. Quantitative and Statistical Analysis

The statistical analyses for the pseudovirus virus neutralization assessments were performed using GraphPad Prism for the calculation of the mean value for each data point. Each specimen was tested in triplicate. Antibody neutralization IC_50_ values were calculated using a five-parameter dose–response curve in GraphPad Prism. For comparison of the serum neutralization titers, statistical analysis was performed using multiple Mann–Whitney tests. Two-tailed *p* values are reported. No statistical methods were used to determine whether the data met the assumptions of the statistical approach.

## 3. Results

### 3.1. Vaccination and Convalescent Sera from COVID-19 Patients Induced ADE of SARS-CoV-2 Pseudoviral Infection on Raji Cells In Vitro

**Figure 1:**
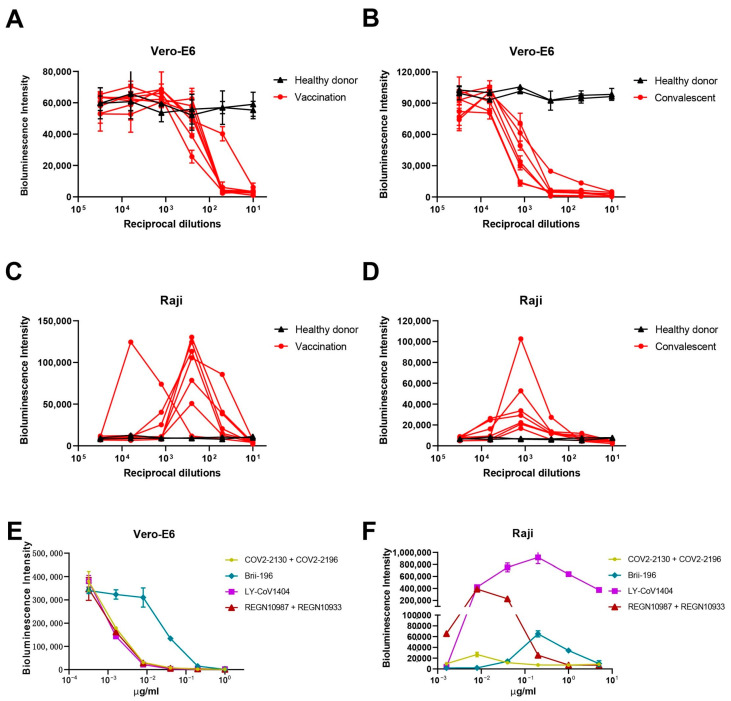
Polyclonal and monoclonal antibodies enhanced SARS-CoV-2 entry into FcR-expressing cells. (**A**) Neutralization of SARS-CoV-2 D614G pseudovirus by sera from seven vaccinated individuals and (**B**) seven convalescent individuals on Vero-E6 cells. (**C**) ADE of SARS-CoV-2 infection by sera from seven vaccinated individuals and (**D**) seven convalescent individuals on Raji cells. (**E**) Neutralization of SARS-CoV-2 D614G pseudovirus by mAbs on Vero-E6 cells. (**F**) ADE of SARS-CoV-2 infection by mAbs on Raji cells. Luciferase activity in the cell lysates was determined at 24 hpi. The experiment was performed in triplicate; means and standard deviations are shown.

We first tested the impact of serum antibodies on SARS-CoV-2 entry into different cells. The SARS-CoV-2 D614G pseudovirus was used to infect the ACE2-expressing Vero-E6 cells or the FcR-expressing Raji cells in the presence of vaccination (*n* = 7), convalescent (*n* = 7), or healthy donor (*n* = 2) sera. All convalescents had reported mild symptoms or were asymptomatic. On Vero-E6 cells, we observed significantly lower luciferase levels in vaccination (Figure 1A, red lines) or convalescent (Figure 1B, red lines) sera compared to healthy control sera (black lines), indicating the viral neutralization activities of the vaccination/convalescent sera, similar to what we previously reported [26,27,28,29]. However, when we tested them on Raji cells, which are cloned B cells highly expressing FcγRIIB [30], we found that both vaccination and convalescent sera (Figure 1C,D, red lines), at certain concentrations, significantly increased luciferase signals compared to control sera (black lines) using the same SARS-CoV-2 D614G pseudovirus. Although the convalescent group had higher neutralization titers than the vaccination group on Vero-E6 cells (Supplementary Figure S1A), the maximum increase in pseudovirus entry of the convalescent group was lower than that of the vaccination group on Raji cells (Supplementary Figure S1B). No correlation was observed between the maximum ADE infectivity fold change and serum neutralization ID_50_ value (*p* = 0.6457, R2 = 0.4845) (Supplementary Figure S1C). These results indicated that polyclonal sera from COVID-19 vaccinees and convalescents could induce ADE of SARS-CoV-2 pseudoviral infection on FcR-expressing cells in vitro.

### 3.2. Approved mAbs Induced ADE of SARS-CoV-2 Pseudoviral Infection on Raji Cells

We next performed similar experiments with mAbs. From a panel of approved SARS-CoV-2 mAbs, Brii-196 (amubarvimab) [31] and LY-CoV1404 (bebtelovimab) [32], as well as two mAb cocktails: COV2-2130 (cilgavimab) + COV2-2196 (tixagevimab) [33], and REGN10987 (imdevimab) + REGN10933 (casirivimab) [34], with high neutralization potency against the prototype virus, were selected. All four mAbs/cocktails could inhibit SARS-CoV-2 entry into Vero-E6 cells (Figure 1E), but when tested on FcR-expressing Raji cells, different degrees of ADE activities were observed. LY-CoV1404 showed the strongest ADE activity, with apparent enhanced infection even at the highest concentration tested (5 μg/mL), while COV2-2130 + COV2-2196 showed much weaker enhancement of SARS-CoV-2 infection compared to other mAbs (Figure 1F). To investigate whether the ADE of SARS-CoV-2 infection could be seen on other immune cells, we tested the same four mAbs/cocktails on viral infection using THP-1, a human monocytic cell line. No ADE activity was observed for all four mAbs/cocktails on THP-1 cells (Supplementary Figure S2), probably due to the much lower FcγRIIB expression level on this cell line [35]. Again, no correlation was observed between the maximum ADE infectivity fold change and mAbs neutralization IC_50_ (*p* = 0.4647, R2 = 0.2865) (Supplementary Figure S1D). Taken together, ADE of SARS-CoV-2 infection can be detected in vitro, but is highly dependent on the FcR expression levels of the target cells.

### 3.3. In Vitro ADE Activities Could Be Eliminated by Adding Human Serum/IgG

**Figure 2:**
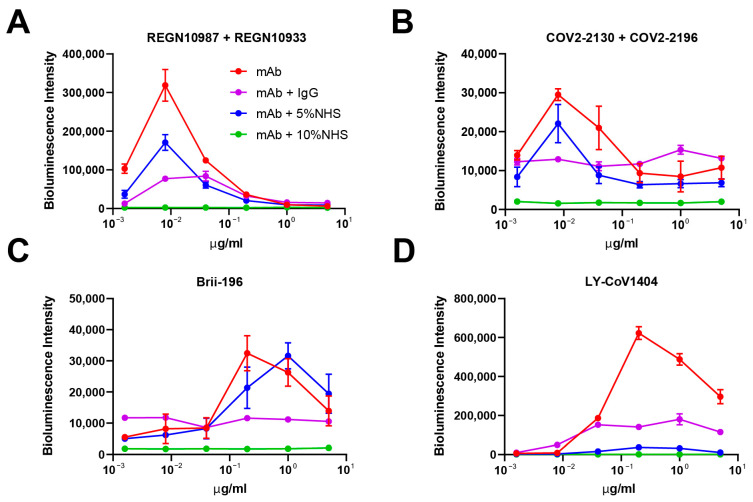
In vitro ADE activities of SARS-CoV-2 neutralizing mAbs could be eliminated by adding human serum/IgG. ADE of SARS-CoV-2 pseudoviral infection on Raji cells mediated by (**A**) REGN10987 + REGN10933, (**B**) COV2-2130 + COV2-2196, (**C**) Brii-196, and (**D**) LY-CoV1404. Cells were cultured without or with human IgG (100 μg/mL), 5% or 10% NHS, and luciferase activity in the cell lysates was determined at 24 hpi. The experiment was performed in triplicate; means and standard deviations are shown.

As our above results indicated that the in vitro ADE relies on antibody Fc interaction with FcRs expressed on immune cells, we then examined whether the ADE Activities would be affected by blocking the Fc–FcR interactions. Serum samples from healthy human adults had been collected before the COVID-19 pandemic and were used as SARS-CoV-2 antibody negative human serum (NHS). First, in order to see the optimal NHS concentration in the cell culture medium sufficient to eliminate mAb-induced ADE activity in vitro, we detected the ADE of LY-CoV1404 on Raji cells cultured in a medium with serially diluted NHS. As the NHS concentration increased, the ADE activity of LY-CoV1404 decreased in a dose-dependent manner and the activity was almost completely abolished when incubated with 5–10% NHS (Supplementary Figure S3). We then measured the ADE of SARS-CoV-2 pseudoviral infection mediated by the four above-tested mAbs/cocktails on Raji cells cultured in a medium with either 5% or 10% NHS. As shown in Figure 2, the enhancement of infection by these antibodies was reduced in the presence of 5% NHS, but was totally abolished in the presence of 10% NHS. We posited that IgG in the serum blocked the interaction of Fc–FcγR and mediated the reduction in ADE. Therefore, we purified human IgG from NHS and performed the experiments in the presence of IgG (100 μg/mL). Weak ADE activities of COV2-2130 + COV2-2196 and Brii-196 could be largely eliminated by IgG, while stronger ADE activities of LY-CoV1404 and REGN10987 + REGN10933 were also greatly reduced (Figure 2). These results indicated that the ADE activities detected in vitro could be eliminated by adding human serum/IgG in the cell culture.

### 3.4. In Vitro ADE Activities Could Be Eliminated by Modification of Antibody Fc Region

**Figure 3:**
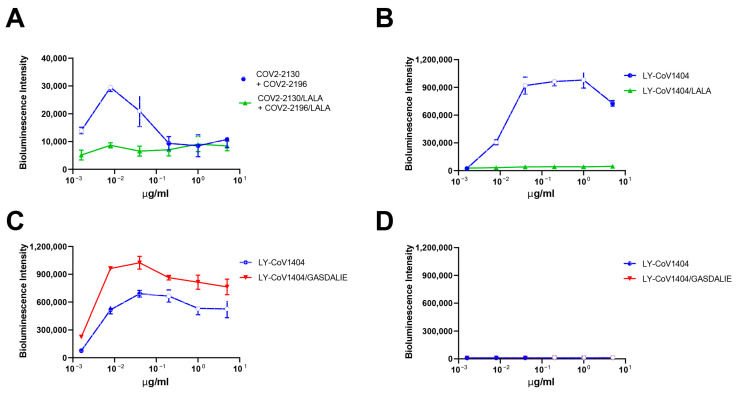
In vitro ADE activities of SARS-CoV-2 mAbs could be abolished by modifying Fc region. ADE of SARS-CoV-2 D614G infection by (**A**) COV2-2130 + COV2-2196 and COV2-2130/LALA + COV2-2196/LALA, (**B**) LY-CoV1404 and LY-CoV1404/LALA, (**C**) LY-CoV1404 and LY-CoV1404/GASDALIE on Raji cells. (**D**) ADE of SARS-CoV-2 XBB.1.5 infection by LY-CoV1404 and LY-CoV1404/GASDALIE on Raji cells. Luciferase activity in the cell lysates was determined at 24 hpi. The experiment was performed in triplicate; means and standard deviations are shown.

To further prove the observed ADE depend on the Fc–FcR interactions, we introduced mutations in the antibody Fc region to abolish its binding to FcRs. L234A/L235A (LALA) mutations, known to abolish Fc-mediated effector functions by preventing mAbs from binding to FcRs but without impacting mAbs’ neutralizing ability [36], were selected. The ADE activity of COV2-2130 + COV2-2196 and LY-CoV1404 was completely abolished by introducing the LALA mutation in the Fc portion of the antibodies (Figure 3A,B). On the other hand, we also generated mAbs with G117A, S120D, A211L, and I213E combined mutations (GASDALIE), known to enhance the antibody-binding affinity to FcγR [37]. As expected, the ADE activity of LY-CoV1404/GASDALIE was further enhanced compared to the WT antibody (Figure 3C). It is noteworthy that when we detected the ADE activity with the most recently emerged Omicron subvariants, XBB.1.5, neither LY-CoV1404 nor LY-CoV1404/GASDALIE showed any ADE activity on Raji cells (Figure 3D). This could be explained by the complete loss of binding and neutralization activity of LY-CoV1404 against XBB.1.5 [38]. These results indicated that the in vitro ADE activities dependent on antibodies’ Fc interaction with FcRs could be abolished by mutating the Fc region.

## 4. Discussion

In this study, we showed that both polyclonal sera from COVID-19 vaccinees and convalescents, as well as approved mAbs for SARS-CoV-2, can induce ADE of pseudoviral infection in vitro. When the vaccination and convalescent sera were tested on Vero-E6 cells that express ACE2, which is the receptor utilized by SARS-CoV-2 for entry, viral neutralization activities were observed, confirming the expected protective effects of the sera. However, on Raji cells that express FcγRIIB, which is a receptor for the Fc portion of antibodies, the sera increased the pseudovirus entry at certain concentrations, indicating the ADE phenomenon. Similarly, approved mAbs for SARS-CoV-2 could also induce ADE of pseudoviral infection on Raji cells. LY-CoV1404 showed the strongest ADE activity, while COV2-2130 + COV2-2196 showed much weaker enhancement of infection compared to other mAbs. No ADE activity was observed on THP-1 cells, probably due to their lower FcγRIIB expression levels compared to Raji cells. This indicates that the susceptibility to ADE activity might be dependent on the FcR expression levels of the target cells. It is worth noting that LY-CoV1404 exhibited the most robust ADE activity with the WT virus on Raji cells, but showed no ADE activity with the XBB.1.5 variant. This indicates that the susceptibility to ADE activity is also influenced by the antibodies’ binding to the virus; therefore, different variants of SARS-CoV-2 may exhibit distinct ADE behaviors. The observed variation in ADE phenomena between vaccinees and convalescents could potentially be attributed to the fact that the vaccination group may have a higher proportion of binding antibodies relative to neutralizing antibodies compared to the convalescent group. As a result, the vaccination group may facilitate greater pseudovirus entry into immune cells at specific concentrations. One limitation of our study is the limited number of donors in each group, particularly the inclusion of only two healthy donors. Nevertheless, our results, together with several other studies [35,39,40,41,42], suggested that we can indeed observe ADE activities of SARS-CoV-2 polyclonal and monoclonal antibodies in certain circumstances in vitro. More importantly, our study is the first to demonstrate that the observed in vitro ADE activities of SARS-CoV-2 can be eliminated by adding human serum or IgG. The enhancement of infection by the tested mAbs was reduced in the presence of 5% human serum, but was totally abolished in the presence of 10% human serum. The addition of purified human IgG also effectively reduced ADE activity, which supports the notion that human IgG in the serum can block the Fc–FcγR interaction and mediate the reduction in ADE. Moreover, this activity can be abolished by introducing mutations in the Fc region that prevent the antibodies from binding to FcRs. As the presence of human IgG in the circulation of individuals provides a natural buffer against ADE activity by blocking the Fc–FcγR interaction, the potential for ADE activity of SARS-CoV-2 sera and mAbs in the real world may not be as high as observed in vitro. The absence of enhanced respiratory diseases or excessive proinflammatory response in SARS-CoV-2 vaccine animal studies and clinical trials [43,44,45], as well as the real-world data involving billions of vaccinated individuals, supports this notion. However, as the number of COVID-19 infections continues to increase globally, particularly with the emergence of new variants, it remains important to monitor the ongoing development of COVID-19 vaccines and neutralizing antibody drugs for potential adverse effects, including ADE. It is crucial to remain vigilant and continue studies to fully understand the role of ADE in human COVID-19 pathology, both in laboratory conditions and in clinical practice.

## 5. Conclusions

In summary, our study highlights that ADE is a complex process that cannot be fully accounted for in vitro. Further research is necessary to assess the occurrence and extent of ADE during actual viral infections. This requires the comprehensive evaluation of preclinical data, as well as clinical trials in humans and animal models. Additionally, monitoring the emergence of new SARS-CoV-2 variants and their potential impact on ADE is essential in ensuring the safety and efficacy of vaccines and antibody therapies.

## Figures and Tables

**Table 1:** List of the primers used in this study.

Primer ^a^	Sequence (5′–3′) ^b^
L234A/L235A-F	TTGTCCCGCCCCTGAGTTTGAGGGCGGACCTTCCGT
L234A/L235A-R	ACGGAAGGTCCGCCCTCAAACTCAGGGGCGGGACAA
G117A/S120D-F	CTGAGCTTCTGGCCGGACCTGATGTGTTCCTGTTCCC
G117A/S120D-R	GGGAACAGGAACACATCAGGTCCGGCCAGAAGCTCAG
A211L/I213E-F	CAACAAGGCCCTGCCCCTACCCGAGGAGAAAACCATCAG
A211L/I213E-R	CTGATGGTTTTCTCCTCGGGTAGGGGCAGGGCCTTGTTG

^a^ F, forward primer; R, reverse primer. ^b^ Antibody sequences were downloaded from PDB.

## Data Availability

All the data are provided in the main body or the supplementary figures.

## Supplementary Materials

The following supporting information can be downloaded at: https://www.mdpi.com/article/10.3390/pathogens12091108/s1, Figure S1: Correlation of the maximum ADE infectivity fold change at which the maximum viral entry with the antibody neutralization ID50; Figure S2: No ADE of SARS-CoV-2 infection by mAbs was observed on THP-1 cells; Figure S3: Determination of the optimal NHS concentration in cell culture medium.
