# Supplementary material for: In Vitro Antibody-Dependent Enhancement of SARS-CoV-2 Infection Could Be Abolished by Adding Human IgG
Source: Pathogens. 2023 Aug 30;12(9):1108. doi: 10.3390/pathogens12091108 (PMC10535176; doi:10.3390/pathogens12091108)
Supplement: Supplementary file 1 [file pathogens-12-01108-s001.zip › pathogens-2537870-supplementary.pdf]

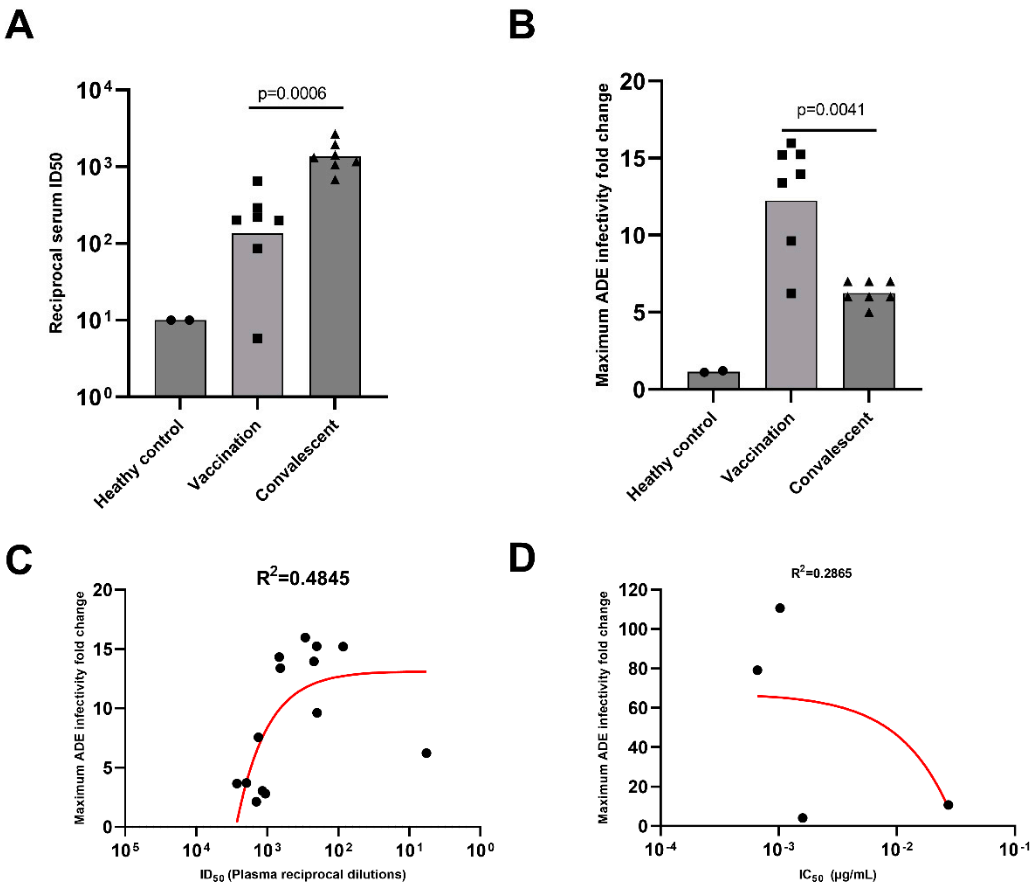

Supplementary Figure S1. Correlation of the maximum ADE infectivity fold change at which the maximum viral entry with the antibody neutralization ID_{50}. (**A**) SARS-CoV-2 D614G neutralization titers of sera from convalescent individuals versus those after 3 doses of inactivated vaccinations. (**B**) Maximum ADE infectivity fold change of sera from convalescent individuals versus those after 3 doses of inactivated vaccinations. (**C**) Correlation of the maximum ADE infectivity fold change at which the maximum viral entry and the serum neutralization ID_{50} for SARS-CoV-2 D614G virus. (**D**) Correlation of the maximum ADE infectivity fold change at which the maximum viral entry with mAbs neutralization IC_{50} for SARS-CoV-2 D614G virus. A linear regression model was used for correlation analysis.

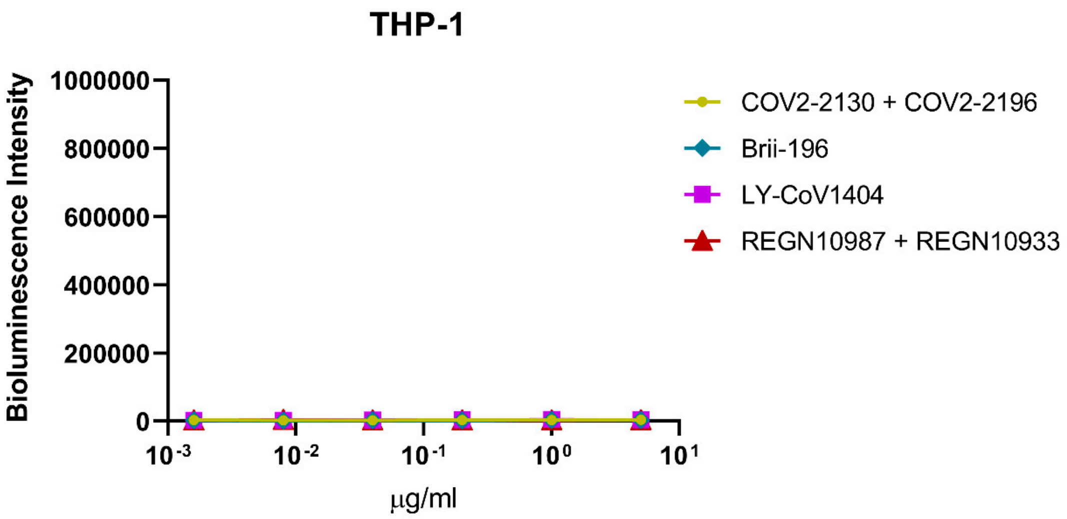

Supplementary Figure S2. No ADE of SARS-CoV-2 infection by mAbs was observed on THP-1 cells.

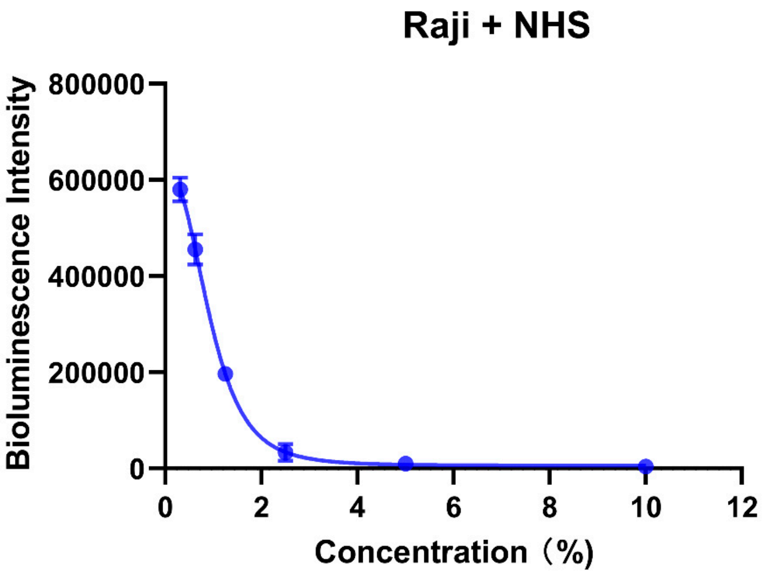

Supplementary Figure S3. Determination of the optimal NHS concentration in cell culture medium that is sufficient to eliminate LY-CoV1404 (1μg/ml) induced ADE activity on Raji cells.
